# Prenatal diethylstilbestrol exposure and risk of diabetes, gallbladder disease, and pancreatic disorders and malignancies

**DOI:** 10.1017/S2040174420000872

**Published:** 2020-10-28

**Authors:** Rebecca Troisi, Marianne Hyer, Linda Titus, Julie R. Palmer, Elizabeth E. Hatch, Dezheng Huo, Kjersti M. Aagaard, William C. Strohsnitter, Robert N. Hoover

**Affiliations:** 1 Department of Health and Human Services, Division of Cancer Epidemiology and Genetics, National Cancer Institute, National Institutes of Health, Bethesda, MD, USA; 2 Information Management Services, Inc., Rockville, MD, USA; 3 Department of Epidemiology, Geisel School of Medicine at Dartmouth and The Norris Cotton Cancer Center, Lebanon, NH, USA; 4 Department of Pediatrics, Geisel School of Medicine at Dartmouth, Lebanon, NH, USA; 5 Slone Epidemiology Unit, Boston University, Boston, MA, USA; 6 Department of Epidemiology, Boston University School of Public Health, Boston, MA, USA; 7 Department of Obstetrics and Gynecology, University of Chicago, Chicago, IL, USA; 8 Department of Obstetrics & Gynecology, Division of Maternal-Fetal Medicine, Baylor College of Medicine and Texas Children’s Hospital, Houston, TX, USA; 9 Department of Molecular, Cell, and Cancer Biology, University of Massachusetts Medical School, Worcester, MA, USA.

**Keywords:** Diethylstilbestrol, prenatal exposure, pancreatitis, pancreatic cancer, diabetes

## Abstract

Prenatal diethylstilbestrol (DES) exposure is associated with increased risk of hormonally mediated cancers and other medical conditions. We evaluated the association between DES and risk of pancreatic cancer and pancreatic disorders, type 2 diabetes, and gallbladder disease, which may be involved with this malignancy. Our analyses used follow-up data from the US National Cancer Institute DES Combined Cohort Study. Cox proportional hazards models estimated hazard ratios (HRs) and 95% confidence intervals (CIs) adjusted for age, sex, cohort, body mass index, smoking, and alcohol for the association between prenatal DES exposure and type 2 diabetes, gallbladder disease (mainly cholelithiasis), pancreatic disorders (mainly pancreatitis), and pancreatic cancer among 5667 exposed and 3315 unexposed individuals followed from 1990 to 2017. Standardized incidence rate (SIR) ratios for pancreatic cancer were based on age-, race-, and calendar year-specific general population cancer incidence rates. In women and men combined, the hazards for total pancreatic disorders and pancreatitis were greater in the prenatally DES exposed than the unexposed (HR = 11, 95% CI 2.6–51 and HR = 7.0, 95% CI 1.5–33, respectively). DES was not associated overall with gallbladder disease (HR = 1.2, 95% CI 0.88–1.5) or diabetes (HR = 1.1, 95% CI 0.9–1.2). In women, but not in men, DES exposure was associated with increased risk of pancreatic cancer compared with the unexposed (HR: 4.1, 95% CI 0.84–20) or general population (SIR: 1.9, 95% CI 1.0–3.2). Prenatal DES exposure may increase the risk of pancreatic disorders, including pancreatitis in women and men. The data suggested elevated pancreatic cancer risk in DES-exposed women, but not in exposed men.

## Introduction

Diethylstilbestrol (DES), a potent synthetic estrogen and endocrine disrupter, was first synthesized in 1938^1^ and shortly thereafter promoted to prevent spontaneous abortion and premature birth.^2^ Over the next three decades, DES was administered to several million pregnant women in the United States and Europe. In 1971, a strong association was reported between prenatal exposure to DES and clear cell adenocarcinoma (CCA) of the vagina and cervix in young women.^3^ This finding was followed by the identification of numerous anatomical anomalies^4^ and adverse reproductive outcomes in prenatally exposed women.^5^

Data from a study conducted by the US National Cancer Institute (NCI) have linked prenatal DES exposure to several cancer outcomes in the female offspring, including vaginal CCA,^6^ cervical neoplasia,^7^ and breast cancer^8^ and suggested an association with testicular cancer in men.^9^ Recent data from the same NCI cohort showed an excess risk of pancreatic cancer among the DES-exposed women compared with general population rates.^10^ The data also suggested a modestly increased risk of type 2 diabetes,^11^ a putative risk factor for pancreatic cancer.^12^ In this paper, we updated our analyses of pancreatic cancer with an additional 5 years of data in the women and for the first time assessed risk of pancreatic cancer in men. We also updated our analyses of diabetes with 11 more years of data (2006–2017). These updates are important given the sharply rising age-incidence curves of these diseases. In addition, for the first time, we examined associations with gallbladder disease and pancreatic disorders, both of which may be involved in the etiology of pancreatic cancer.^13,14^

While DES is no longer used in pregnancy and the individuals who were historically exposed *in utero* are aging, the influence of prenatal exposure to other environmental estrogens remains highly relevant. Measuring prenatal exposure to environmental estrogens and assessing their long-term health effects are extremely challenging. Our DES cohorts, which have documented prenatal exposure and decades of follow-up, offer important insights into the possible long-term effects of other endocrine disruptors on adult health outcomes. Animal and epidemiological studies of Bisphenol A (BPA), a weaker but ubiquitous and similar synthetic estrogen,^15,16^ suggest links with disorders in glucose metabolism^17,18^ and cancer.^19^ The data from our cohorts, based on known prenatal exposure to a prototypical endocrine disruptor, provide a model to calibrate concerns about the influence of current environmental estrogens on health.

## Materials and methods

Approvals for the study were obtained from the investigational review boards at the study sites and the NCI. Participants indicated their informed consent by completion of a questionnaire or telephone interview and by signed medical record release forms.

### Cohorts

In the early 1990s, the US NCI assembled new and previously followed cohorts of exposed and unexposed women and men for combined follow-up. The NCI DES Combined Cohort Follow-up Study consists of prenatally exposed and unexposed: (1) women who participated in the National Cooperative Diethylstilbestrol Adenosis Project ([DESAD] cohort);^20^ (2) men whose mothers were patients at the Mayo Clinic for the period from 1939 and 1962;^21^ (3) women and men whose mothers participated in a clinical trial of DES in 1951–1952 (Dieckmann Cohort);^22^ (4) women and men whose mothers were treated in a large private infertility practice in Massachusetts, USA (Horne Cohort), and (5) women and men from Massachusetts, New Hampshire, and Maine whose mothers participated in the Women’s Health Study ([WHS] Cohort).^23^ The follow-up of the combined cohorts began in 1994 with a mailed questionnaire, and questionnaires subsequently were mailed at approximately 5-year intervals beginning in 1997, 2001, 2006, 2011, and 2016 (administration of the 2016 questionnaire was delayed in one of the DESAD subcohorts [Texas]; for this subcohort, data through the 2011 follow-up are included in the current analysis).

### Participants

A total of 6571 (4474 exposed, 2097 unexposed) women were eligible for enrollment in the NCI DES Combined Cohort Follow-up Study. Women who participated in NCI follow-up (i.e. completed at least one of the combined follow-up questionnaires), or who were eligible for participation and died of one of the outcomes, were included in the current analysis. The response rates to the questionnaire ranged from 94% of the exposed and 96% of the unexposed for the 1994 questionnaire to 73% and 74%, respectively, for the 2016 questionnaire. Of the 6048 (4214 exposed and 1834 unexposed) women included in the analysis, 222 died before the end of follow-up, and 3537 were followed through 2017; the remainder were censored at their last follow-up.

A total of 3600 (1710 exposed, 1890 unexposed) men were eligible for enrollment in the NCI study. Men who participated in the NCI follow-up (i.e. completed at least one of the combined follow-up questionnaires), or who were eligible for participation and died of one of the outcomes, were included in the current analysis. The response rates to the questionnaire ranged from 93% in the exposed and 92% in the unexposed for the 1994 questionnaire to 75% and 73%, respectively, for the 2016 questionnaire. Of the 2934 (1453 exposed and 1481 unexposed) men included in the analysis, 256 died before the end of follow-up, and 1971 were followed through 2017; the remainder were censored at their last follow-up.

### Ascertainment of pancreatic and gallbladder cancer

All questionnaires queried participants on cancer diagnoses, the year of diagnosis, and its anatomic origin. Pathology records were retrieved to confirm incident cancer diagnoses reported on the questionnaires. For cohorts originating in California, Massachusetts, Minnesota, New Hampshire, and Texas, cancer or tumor (Mayo Clinic) registries (approximately 88% of participants) were periodically searched to obtain additional information on reported cases and to identify new cases. Cases were also identified from death certificates and International Classification of Diseases (ICD) coding of underlying and contributing causes of death from the National Death Index (NDI) Plus, which was routinely searched for participants who were lost to follow-up or had an unknown cause of death, and for individuals who were eligible to participate in the NCI study but did not return a questionnaire.

A total of 11 pancreatic cancers were reported on the questionnaires (1 by proxy). Of these 11, pathology reports were obtained for 7 and confirmed the diagnosis, 1 was confirmed by NDI-Plus, and for the remaining 3 reported cases no supporting evidence was retrieved. In the absence of pathology or registry/NDI confirmation, pancreatic cancer outcomes were based on self-reported diagnoses (*n* = 3). We identified three additional cases from state cancer registries and nine additional cases from NDI-Plus. The nine cases ascertained through NDI-Plus included three deaths among individuals who did not return an NCI questionnaire. In total, the analysis included 23 pancreatic cancer cases (15 in women and 8 in men). Of the pancreatic cancers identified by pathology reports and cancer registries (ICD-9 = 157, ICD-10 = C25), eight were adenocarcinoma, one was invasive islet cell tumor, and one was acinar cell carcinoma; the pancreatic cancer cases identified by NDI-plus (*n* = 9) could not be classified as exocrine or endocrine tumors (all were coded ICD-9 = 157.9 or ICD10 = C25.9).

Invasive gallbladder and biliary tumors (ICD-9 = 156) were also identified. There were only two cases; a gallbladder tumor in an exposed woman and a biliary tumor in an unexposed man.

### Ascertainment of nonmalignant pancreatic disorders, gallbladder disease, and type 2 diabetes

Nonmalignant pancreatic disorders and gallbladder disease were identified through responses to two open-ended questions. The first of these questions was asked on the 1994 questionnaire, *“Have you had any other serious medical conditions requiring hospitalization, surgery or continuing treatment in the past 5 years?”* and further queried participants for the year of diagnosis or hospitalization. The 1997 questionnaire included the same question but queried the time period since the date of last questionnaire response. The 2001, 2006, 2011, and 2016 questionnaires included the following question: *“Since (year of last response), have you ever been diagnosed with any of the following conditions by a health professional?”* The response options, offered in a checklist, included adult-onset diabetes (henceforth referred to as type 2 diabetes), high cholesterol, hypertension, coronary artery disease, myocardial infarction, stroke, osteoporosis, and fractures, previously analyzed with respect to DES exposure.^11^ The questionnaires also included an open-ended question: “Other, specify:” allowing participants to report unlisted outcomes.

Responses to open-ended questions were coded by two nosologists using ICD-9 and blinded to DES exposure status; discrepancies were arbitrated by a supervisor. This process identified a total of 24 benign pancreatic disorders (ICD-9 = 577, *n* = 17 pancreatitis and *n* = 7 other pancreatic conditions including intraductal papillary mucinous neoplasia; pancreatic splenectomy for benign tumor; pancreas infection; cysts on pancreas; pancreas blockage; pancreatic insufficiency; birth defect in pancreas). In addition, 275 participants reported gallbladder or biliary disease (ICD-9 = 574–576). An additional two cases of benign biliary disease were identified from NDI-Plus.

Type 2 diabetes diagnoses were identified through the checklist responses (*n* = 708) or as an underlying or contributing cause of death from NDI-Plus (*n* = 29). The diabetes cases ascertained through NDI-Plus included 12 deaths among individuals who were eligible to participate but did not return an NCI questionnaire.

### DES exposure and covariate ascertainment

For all combined cohort participants, prenatal exposure to DES, or the lack thereof, as well as indication (if one existed) for use, was documented by the medical record or a physician’s note. Gestational week of first DES use was available for 75% of exposed women. Because data on total cumulative DES dose were available for only 38% of the women, we classified the individual cohorts as high- or low-dose based on differences in prescribing practices by US region (unknown for a subgroup of the WHS). Agreement between the dose categories and individual doses among those with complete data was excellent.^8^ Lifetime history of education (highest grade completed), cigarette smoking (ever smoked cigarettes regularly for 6 months or longer; current and former status with date of cessation in the latter), ever use of alcohol (at least one alcoholic beverage per month for 6 months or longer), ever use of hormone replacement therapy, as well as information on body size (height and weight) and frequency of routine medical examinations in the last 5 years were collected on the 1994 questionnaire. Smoking, alcohol, body weight, and routine medical screening were updated on subsequent questionnaires (in 2006 and 2016 for smoking, in 2016 for alcohol, and in 2006, 2011, and 2016 for body weight). Body mass index (BMI; weight (kg)/height (m)^2^) was calculated. The Dieckmann and DESAD studies incorporated a comprehensive gynecologic examination around the time of recruitment that systematically identified vaginal epithelial changes (VEC), a marker of host susceptibility, by means of colposcopy or iodine staining. These changes were more frequent in women exposed to DES early in pregnancy who also had large cumulative doses of DES by the end of pregnancy.^24^

### Statistical analysis

We analyzed the associations of prenatal DES exposure with diabetes, gallbladder disease, pancreatic disorders, and pancreatic cancer. Gallbladder/biliary cancers were not statistically analyzed because there were only two cases. Follow-up began in 1990 because the baseline questionnaire ascertained outcomes from that point forward, and person-years were accrued until the earliest of the following dates: diagnosis of or death from the reported outcome or return of the participant’s most recent questionnaire.

Associations between prenatal DES exposure and outcomes of interest were estimated with hazard ratios (HRs) and 95% confidence intervals (CIs) using Cox proportional hazards regression models.^25^ The models included terms for original cohort, sex and birth year and used age as the underlying time parameter. Models were repeated among women and men separately. Multivariable models additionally included terms for BMI categories and smoking status (based on both the 1994 data only and on the time-dependent variables that included information from subsequent questionnaires) and alcohol use (updated as of the 2016 questionnaire). The categories for each of the covariates are those listed in Table 1. Only 7% of exposed and 4% of unexposed women and 6% of both exposed and unexposed men had any missing values for covariates. Missing values were categorized separately and included in the models; a complete case approach, which excluded those who were missing covariate data, was performed as a sensitivity analysis. The assumption of proportional hazards was confirmed in models containing a term for the interaction between DES and attained age (as a time-dependent variable).

In analyses confined to the exposed, we evaluated outcomes related to DES characteristics, including DES dose and timing of first DES exposure (excluding the subcohort of the WHS in which dose was unknown), and in the Dieckmann and DESAD cohorts, analyses in women were carried out by the absence or presence of VEC.

For the pancreatic cancer analysis, we also performed external comparisons based on age- and calendar-year-specific cancer incidence rates from the Surveillance, Epidemiology and End Results Program (SEER; National Cancer Institute SEER*Stat software (www.seer.cancer.gov/seerstat) version 8.3.6.)^26^ for white women and men from 1973 to 2016. SEER incidence rates include both exocrine and endocrine pancreatic tumors. We computed SIR ratios and their 95% CI assuming a Poisson distribution for the observed case numbers.

## Results

Most exposed and unexposed women and men were from the DESAD and Mayo cohorts, respectively (Table 1). There were small differences in the characteristics of the DES-exposed and unexposed women. Exposed women were younger, had more years of education, were less likely to smoke than the unexposed, but were similar in their reported alcohol intake. BMI was also generally similar by DES exposure at baseline (Table 1) and throughout follow-up (data not shown). Frequency of general physical examinations in the last 5 years as reported on the 1994 questionnaire was similar in the exposed and unexposed women. Among the women, 2.9% of the exposed and 3% of the unexposed reported a family history of pancreatic cancer. Exposed men were slightly older and more educated but similar to unexposed men with regard to smoking, alcohol use, and frequency of physical exams. Among men, BMI was similar by DES exposure at baseline and throughout follow-up (data not shown). Among men, 2.4% and 2.6% of the exposed and unexposed, respectively, reported a family history of pancreatic cancer.

Among women, 48% of the exposed and 46% of the unexposed reported at least one medical condition in the open-ended questions after excluding conditions related to DES (i.e. infertility, pregnancy complications, cervical dysplasia, and breast cancer). Among women who reported any condition in the open-ended questions, the average number of conditions reported was comparable in the exposed and unexposed (2.1 for exposed and 2.0 unexposed). Among men, 42% of the exposed and 41% of the unexposed reported at least one medical condition in the open-ended questions, and among those who reported any condition, the average number of reports was comparable in the exposed and unexposed (2.0 for exposed and 1.9 unexposed).

The associations between prenatal DES exposure and the outcomes of interest are presented in Table 2. DES was not associated with risk of diabetes (HR 1.1, 95% CI 0.90–1.2) or gallbladder disease (HR 1.2, 95% CI 0.88–1.5) overall or in sex-specific groups. Risk of pancreatic disorders was highly elevated overall (HR 11, 95% CI 2.6–51) and among both women and men, although case numbers were limited among the unexposed, and CI were wide. Overall risk remained elevated when pancreatic disorders were confined to pancreatitis (HR 7.0, 95% CI 1.5–33); risks were also elevated in sex-specific analyses, but estimates were imprecise. The HR for DES and pancreatic cancer was 1.6, 95% CI 0.62–4.1 overall and was elevated among women (4.1, 95% CI 0.84–20), but estimates were imprecise. There was no evidence of increased risk of pancreatic cancer in DES-exposed men (HR 0.50, 95% CI 0.11–2.2).

Adjustment for covariates only minimally changed the estimates between DES exposure and pancreatic disorders and pancreatic cancer. Estimates from complete case analyses were largely similar to estimates from models that included a missing category, although the HR for pancreatic cancer in women and men combined was attenuated. The HR for pancreatic cancer among women and men was 1.2 (95% CI 0.43–3.1) based on a complete case approach and 1.6 (95% CI 0.62–4.1) using models with a missing category; the respective estimates in women were 3.7 and 4.1. The results in men were similar for both approaches (data not shown). After excluding two DES-exposed women and 1 unexposed man whose self-reported pancreatic cancer was unconfirmed, the HR was 1.7 (95% CI 0.62–4.7) overall, 3.3 (95% CI 0.66–16.3) in women and 0.78 (95% CI 0.17–3.6) in men. Additional adjustment for ever taking hormone replacement therapy did not change the HRs for any of the outcomes in the women (data not shown). HRs for the association of DES exposure with pancreatic cancer risk also were unchanged when time-dependent variables representing cigarette smoking or BMI replaced the baseline measures of those factors (data not shown).

In external comparisons with the general population, the overall SIR for pancreatic cancer for the DES exposed was 1.4 (95% CI 0.82–2.3) based on 16 observed cases and 0.86 (95% CI 0.35–1.8) for the unexposed based on seven observed cases. Among exposed women, the SIRs were 1.9 (95% CI 1.0–3.2) based on all 13 cases and 1.6 (95% CI 0.80–2.9) based on 11 confirmed cases. Among the unexposed women, the SIR was 0.53 (95% CI 0.06–1.9) based on only two cases. Among the exposed men, the SIR was 0.70 (95% CI 0.14–2.1) based on three cases. Among the unexposed men, the SIRs were 1.2 (95% CI 0.37–2.7) based on five cases and 0.92 (95% CI 0.25–2.4) based on four confirmed cases.

Analyses assessing outcomes associated with DES exposure characteristics (timing of first exposure; dose level; and in women, VEC status) were confined to individuals who were DES exposed. In these analyses, results for the outcomes of interest were generally similar for characteristics of DES exposure. The few differences noted may be due to the restriction of the analysis to the DESAD and Dieckmann cohorts, for whom these data were available, and consequent reduction in sample sizes (Table 3). Among the DES exposed, diabetes risk was lower in those who were exposed to DES early in gestation (<8 weeks and 8–12 weeks and combined into <13 weeks), compared to later (13+ weeks), both overall (HR 0.79, 95% CI 0.63–1.0) and in women (HR 0.74, 95% CI 0.56–0.98). Risk of type 2 diabetes was also lower in exposed women with VEC compared to those without VEC (HR 0.80, 95% CI 0.62–1.0). There was no evidence that timing or dose of prenatal DES exposure influenced risk of gallbladder disease in women. Although the HR was elevated for gallbladder disease among the men who were exposed to DES early in gestation, the CIs were wide (HR 1.7, 95% CI 0.48–5.8). Risk of total pancreatic disorders was elevated in those exposed to a high cumulative DES dose compared with a low dose (HR 7.0, 95% CI 1.9–26); this was also observed in analyses restricted to women (HR 3.6, 95% CI 0.98–13). All the cases among men were exposed to a high cumulative dose.

## Discussion

We observed elevated risk of pancreatic disorders among women and men who were prenatally exposed to DES compared with those who were not. Although the risk estimates were imprecise, the positive association between DES and pancreatic disorders remained after adjustment for covariates, such as BMI, smoking, and alcohol use, and was more pronounced in women with VEC, a marker for DES host susceptibility, compared with those without VEC.

The current analysis extends our prior findings regarding adverse medical conditions^11^ by examining prenatal DES exposure in relation to pancreatic disorders, the majority of which (71%) were pancreatitis. Our data indicate an 11-fold increased risk of any pancreatic disorder and a 7-fold increased risk of pancreatitis. The higher estimate for any pancreatic disorder could reflect overreporting by the DES exposed, perhaps due to greater general health concerns, although that seems unlikely. DES is known to be associated with reproductive outcomes and possibly with some cancers but has not been identified as a risk factor for pancreatic disorders. The risk estimates for gallbladder disease, a condition of similar severity, were not elevated, suggesting that general overreporting by the DES exposed is unlikely. Also, the average number of conditions reported and the percentage of participants reporting at least one medical condition in the open-ended “other,” category included in the questionnaire checklist of specific conditions was similar for the exposed and unexposed participants. Consequently, if self-reported health outcomes in our data were misclassified, the misclassification is likely to be nondifferential, which would bias an association toward the null.

Greater surveillance of DES-exposed participants, and possibly more prevalent imaging, also could have resulted in more opportunities for diagnosis, especially of asymptomatic pancreas anomalies, but the frequency of routine physical exams was similar among the DES exposed and unexposed. Also, acute pancreatitis (AP) is a serious and sometimes fatal condition that typically presents with abdominal pain and is diagnosed based on clinical symptoms and elevations in serum amylase or lipase. Annual incidence of AP in the United States has been reported to range from 4.9 to 35 per 100,000 population,^27^ with most cases due to gallstones or alcohol-induced injury. Smoking also may increase the risk for pancreatitis.^28^ Assessing the prevalence of chronic pancreatitis (CP) is more challenging but in one study was estimated to be 100/100,000 in US adults over a 13-year period.^29^ Approximately 20% of patients with AP have a recurrence and 36% of those with recurrent AP develop CP.^30^ Thus, the development of AP, recurrent AP, and ultimately CP is thought to represent a nonobligate disease continuum.^31^ Overall incidence of reported pancreatitis (only one event per participant) in our cohort was approximately 8.3/100,000 (17/204,855 person-years) which could reflect their relatively low rates of smoking and alcohol use. Adjustment for alcohol use and smoking status did not affect the risk estimates for DES and pancreatitis.

Prenatal DES exposure was not associated with gallbladder disease in our study. Also, adjustment for BMI did not affect the association of DES and pancreatic disorders. Considered together, these findings suggest that any possible causal association of DES with pancreatic disorders is not mediated by effects of gallbladder disease or pathological mechanisms relating to obesity.

Type 2 diabetes is a symptom of, and prognostic factor for, pancreatic cancer and may also be involved in its etiology.^32^ In mice, prenatal DES exposure is associated with elevated circulating levels of insulin.^33^ Also, in mice, prenatal exposure to BPA, a similar but less potent estrogen than DES, is associated with alterations in glucose homeostasis and endocrine pancreatic function in prenatally exposed offspring.^17^ We previously reported that prenatal DES exposure was associated with a modestly increased risk of diabetes in women (HR 1.5, 95% CI 1.0–2.1) but not in men. In the current analysis, which adds approximately 10 years of follow-up, the HR among the women was attenuated (HR 1.2, 95% CI 0.94–1.5). As before, there was no evidence of a positive association of prenatal DES exposure with risk of diabetes in men.

Our previous analysis indicated a strong association between prenatal DES exposure and pancreatic cancer overall, but small case numbers precluded separate analyses in women and men.^10^ In the current data, a fourfold increased risk was observed in DES-exposed women, but CIs were wide, and the finding was compatible with chance. When restricted to confirmed cases, the association was slightly attenuated (HR = 3.3). Pancreatic cancer was not elevated, and possibly reduced, in DES-exposed men. Approximately 50% of pancreatic cancers were initially identified by self-report on the questionnaires, but most were verified, and results did not differ dramatically in analyses confined to verified cases. Like others,^34,35^ we have found generally high validation for self-reported cancers other than non-melanoma skin cancer and cervical cancer.^36^ While the DES exposed are aware of associations with reproductive cancers and breast cancer, it is unlikely that there would be differential reporting of pancreatic cancer by DES exposure. With the low survival rate associated with these tumors and time between questionnaires (about 5 years), underascertainment, however, is possible, especially as we did not have complete coverage through the cancer registry search effort. Ascertainment of cases through NDI and cancer registries, however, is not likely to differ by DES exposure status.

In summary, prenatally DES-exposed women and men may have an elevated risk of pancreatitis and other pancreatic disorders, and in women, prenatal exposure to DES may increase risk of pancreatic cancer. If the association with pancreatic disorders is causal, the effect of DES does not appear to involve pathways that include gallbladder disease or type 2 diabetes.

## Figures and Tables

**Table 1.** Characteristics of prenatally DES-exposed and unexposed study participants

Characteristic	*Women*				*Men*			
	Exposed		Unexposed		Exposed		Unexposed	
	*N*	%	*N*	%	*N*	%	*N*	%
	4214	100	1834	100	1453	100	1481	100

*Cohort*								

DESAD/Mayo^a^	3443	82	868	47	674	46	619	42

Dieckmann	287	7	253	14	265	18	244	16

Horne	209	5	145	8	262	18	180	12

WHS	275	7	568	31	252	17	438	30

*Birth year*								

<1950	692	16	460	25	465	32	388	26

1950–1954	1792	43	773	42	582	40	631	43

1955–1959	1063	25	430	23	166	11	288	19

1960 +	667	16	171	9	240	17	174	12

*Education (1994)*								

≤High school	553	13	379	21	231	16	291	20

Some college	915	22	447	24	270	19	341	23

4-year college	1423	34	561	31	508	35	406	27

Graduate school	1098	26	408	22	326	22	321	22

Missing	225	5	39	2	118	8	122	8

*Smoking status (1994)*								

Never	2333	55	905	49	691	48	694	47

Ever	1645	39	881	48	693	48	709	48

Missing	236	6	48	3	69	5	78	5

*Alcohol intake (2016)*								

No	770	18	331	18	138	9	147	10

Yes	3273	78	1466	80	1273	88	1285	87

Missing	171	4	37	2	42	3	49	3

*Body mass index (1994)*								

<20	606	14	267	15	23	2	24	2

20–24	2091	50	905	49	511	35	479	32

25–29	772	18	386	21	623	43	650	44

>30	493	12	219	12	235	16	254	17

Missing	252	6	57	3	61	4	74	5

*General physical exams (in last 5 years; 1994)*								

0	612	15	230	13	251	17	258	17

1	976	23	406	22	499	34	449	30

2–3	1301	31	600	33	456	31	510	34

4+	1024	24	517	28	169	12	179	12

Missing	301	7	81	4	78	5	85	6

Percentages in table do not always add to 100.0 because of rounding.

a DESAD for Women’s Cohort; Mayo for Men’s Cohort.

**Table 2.** Hazard ratios (HRs) and 95% confidence intervals (CIs) for prenatal DES exposure and type 2 diabetes, gallbladder disease, pancreatic disorders, and pancreatic cancer

	Prenatal DES status				HR^a^	95% CI	HR^b^	95% CI
	Exposed		Unexposed					
	Cases	Person-years	Cases	Person-years				

*Total*								

Diabetes	444	124,500	293	73,370	1.1	0.90–1.2	1.1	0.90–1.2

Gallbladder disease	188	125,819	89	74,820	1.2	0.88–1.5	1.2	0.88–1.5

Pancreatic disorders	22	128,611	2	76,179	12	2.6–52	11	2.6–51

Pancreatitis only	15	128,676	2	76,179	7.1	1.5–33	7.0	1.5–33

Pancreatic cancer	16	128,897	7	76,193	1.6	0.63–4.1	1.6	0.62–4.1

*Women*								

Diabetes	288	92,553	118	41,018	1.3	1.0–1.6	1.2	0.94–1.5

Gallbladder disease	168	92,679	67	41,156	1.2	0.87–1.6	1.2	0.87–1.6

Pancreatic disorders	16	95,303	1	42,299	13	1.5–102	12	1.5–96

Pancreatitis only	12	95,342	1	42,299	8.8	1.0–75	8.5	0.99–72

Pancreatic cancer	13	95,489	2	42,297	4.1	0.84–20	4.1	0.84–20

*Men*								

Diabetes	156	31,943	175	32,352	0.90	0.72–1.1	0.94	0.75–1.2

Gallbladder disease	20	33,140	22	33,664	1.0	0.54–1.8	1.0	0.56–1.9

Pancreatic disorders	6	33,308	1	33,879	9.6	1.1–84	10	1.2–86

Pancreatitis only	3	33,334	1	33,879	4.5	0.45–44	4.4	0.45–44

Pancreatic cancer	3	33,407	5	33,900	0.59	0.14–2.5	0.50	0.11–2.2

a Adjusted for birth year, (sex) and cohort.

b Adjusted for birth year, (sex), cohort, body mass index, smoking status, and alcohol use.

**Table 3.** Hazard ratios (HRs)^a^ and 95% confidence intervals (CIs) for type 2 diabetes, gallbladder disease, pancreatic disorders, and pancreatic cancer in DES exposed for timing of first prenatal DES exposure^b^ and DES dose^c^ and presence or absence of vaginal epithelial changes (VEC) in women^d^

	Total				Women				Men			
	Cases	Person-ears	HR^a^	95% CI	Cases	Person-years	HR^a^	95% CI	Cases	Person-years	HR^a^	95% CI

*Diabetes*												

<13 weeks	198	63,325	0.79	0.63–1.0	128	47,053	0.74	0.56–0.98	70	16,272	0.90	0.61–1.3

13+ weeks	132	29,051	1.0	–	85	21,743	1.0	–	47	7308	1.0	–

High dose	226	70,453	0.95	0.77–1.2	167	56,145	0.90	0.70–1.2	59	14,307	1.1	0.78–1.6

Low dose	192	46,937	1.0	–	106	32,426	1.0	–	86	14,511	1.0	–

VEC					116	40,392	0.80	0.62–1.0				

No VEC					137	39,917	1.0	–				

*Gallbladder disease*												

<13 weeks	90	63,601	1.1	0.75–1.6	82	46,835	1.0	0.70–1.5	8	16,766	1.7	0.48–5.8

13+ weeks	44	29,732	1.0	–	40	21,964	1.0	–	4	7768	1.0	–

High dose	107	70,809	1.1	0.82–1.5	100	56,114	1.2	0.83–1.6	7	14,692	1.0	0.36–2.8

Low dose	70	47,669	1.0	–	58	32,482	1.0	–	12	15,188	1.0	–

VEC					69	40,384	0.93	0.67–1.3				

No VEC					74	39,932	1.0	–				

*Pancreatic disorders*												

<13 weeks	10	65,053	1.5	0.44–4.9	8	48,218	1.3	0.37–4.4				

13+ weeks	4	30,370	1.0	–	4	22,558	1.0	–				

High dose	16	72,488	**7.0**	**1.9–26**	12	55,732	3.6	0.98–13				

Low dose	3	48,697	1.0	–	3	33,383	1.0	–				

VEC					9	41,462	2.1	0.66–6.5				

No VEC					5	41,106	1.0	–				

*Pancreatic cancer*												

<13 weeks	7	65,142	0.65	0.22–1.9	7	48,291	0.96	0.29–3.2				

13+ weeks	7	30,455	1.0	–	5	22,633	1.0	–				

High dose	10	72,718	2.0	0.63–6.6	8	57,890	1.5	0.44–5.4				

Low dose	5	48,724	1.0	–	4	33,410	1.0	–				

VEC					5	41,570	1.0	0.30–3.5				

No VEC					6	41,171	1.0	–				

a HRs are adjusted for birth year and cohort (except dose); number of cases of pancreatic disorders and cancer was insufficient to present for men.

b Timing excludes the WHS cohort in which data were unavailable for gestational age at first use.

c Dose is based on cohort and excludes participants from New Hampshire among whom dose was unavailable. High dose includes Dieckmann, DESAD (Boston, California), Horne, and WHS(Boston); low dose includes DESAD (Minnesota, Wisconsin, Texas) and Mayo (Men). Dose models were not adjusted for cohort.

d VEC was available for women in Dieckmann and DESAD exposed cohorts only.
